# H3S28P Antibody Staining of Okinawan *Oikopleura dioica* Suggests the Presence of Three Chromosomes

**DOI:** 10.12688/f1000research.25019.2

**Published:** 2021-03-01

**Authors:** Andrew W. Liu, Yongkai Tan, Aki Masunaga, Aleksandra Bliznina, Charlotte West, Charles Plessy, Nicholas M. Luscombe

**Affiliations:** 1 Genomics and Regulatory Systems Unit, Okinawa Institute of Science and Technology, Graduate University, Onna-son, Okinawa, 904-0324, Japan; 2 Francis Crick Institute, London, NW1 1AT, UK; 3 Department of Genetics, Evolution and Environment, University College London, London, WC1E 6BT, UK

**Keywords:** karyotype, chromosome, centromere, histone H3, Oikopleura, oocyte, embryo, H3S28P

## Abstract

*Oikopleura dioica* is a ubiquitous marine zooplankton of biological interest owing to features that include dioecious reproduction, a short life cycle, conserved chordate body plan, and a compact genome. It is an important tunicate model for evolutionary and developmental research, as well as investigations into marine ecosystems. The genome of north Atlantic *O. dioica *comprises three chromosomes. However, comparisons with the genomes of *O. dioica *sampled from **mainland and southern Japan revealed extensive sequence differences. Moreover, historical studies have reported widely varying chromosome counts. We recently initiated a project to study the genomes of *O. dioica *individuals collected from the coastline of the Ryukyu (Okinawa) Islands in southern Japan. Given the potentially large extent of genomic diversity, we employed karyological techniques to count individual animals’ chromosomes *in situ* using centromere-specific antibodies directed against H3S28P, a prophase-metaphase cell cycle-specific marker of histone H3. Epifluorescence and confocal images were obtained of embryos and oocytes stained with two commercial anti-H3S28P antibodies (Abcam ab10543 and Thermo Fisher 07-145). The data lead us to conclude that diploid cells from Okinawan *O. dioica *contain three pairs of chromosomes, in line with the north Atlantic populations. The finding facilitates the telomere-to-telomere assembly of Okinawan *O. dioica *genome sequences and gives insight into the genomic diversity of *O. dioica* from different geographical locations. The data deposited in the EBI BioImage Archive provide representative images of the antibodies’ staining properties for use in epifluorescent and confocal based fluorescent microscopy.

## Introduction

The larvacean, *Oikopleura dioica*, possesses a fascinating genome: it has reduced to a mere 70Mbp and exhibits unique characteristics such as non-canonical splicing and the scattering of Hox genes ( Denoeud *et al.*, 2010; Edvardsen *et al.*, 2005; Marz *et al.*, 2008; Seo *et al.*, 2001). It is thought that a combination of large effective population size and high mutation rate per generation have led to fast evolution ( Berná & Alvarez-Valin, 2014). The recently published genome sequence of a “Japanese *O. dioica”* from mainland Japan highlighted large sequence variations between the Pacific and Atlantic populations ( Wang *et al.*, 2020). In addition, we recently released a telomere-to-telomere genome sequence of an *O. dioica* individual collected from the Okinawan coastline in southern Japan ( Bliznina *et al.*, 2020), which, to our surprise, revealed large differences in synteny to the mainland Japanese genome despite the geographical proximity. The genetic map of the north Atlantic *O. dioica* is reported to contain three chromosomes (two autosomes, X and Y sex chromosomes; Denoeud *et al.*, 2010); however, prior studies based on histochemical techniques reported three ( Körner, 1952) and eight chromosomes ( Colombera & Fernaux, 1973). Given the large sequence and synteny differences between the assembled *O. dioica* genomes, as well as the discrepancies among previous studies, we wished to assess the karyotype for the local Okinawan *O. dioica* population.

Karyotyping is a long-established histochemical method to visualize eukaryotic chromosomes ( Hsu & Benirschke, 1967; Tjio & Levan, 1950). This rapid technique, involving the use of stains including methylene blue, eosin, and azure B, allows for observation of chromosomes with a simple light microscope, naturally lending itself to a first attempt for karyotyping analysis ( Giemsa, 1904). However, we were unable to determine an accurate count for the Okinawan *O. dioica* by this method due to variability which ranged from 11–27 chromosomes per nucleus.

As an alternative approach, we decided to immunostain the centromere as a means of quantifying the number of chromosomes. Metaphase-specific histone 3 (H3) markers have been used to determine the structure and the segregation of genetic material during oogenesis in situ ( Ganot *et al*., 2006; Schulmeister *et al.*, 2007). One such marker that has been successfully visualized in *O. dioica* is histone H3 phosphorylated at Ser-28 ( Kawajiri *et al.*, 2003; Kurihara *et al.*, 2006), whose localization depends on the phase of the cell cycle: during metaphase, sister chromatids were stained in a manner consistent with alignment along the metaphase plate, whereas in non-mitotic cells, spatially punctate signals were found evenly spread within the nuclear envelope ( Campsteijn *et al.*, 2012; Feng & Thompson, 2018; Feng *et al.*, 2019; Olsen *et al.*, 2018). A structure in which chromosomes are sequestered in a ∏-shaped conformation has also been observed during meiotic cell divisions between the final phases of oogenesis and mature oocytes ( Ganot *et al.*, 2008). In Table 1, we list the publications in which the H3S28P marker was applied to *O. dioica*: the studies were all performed using cultured strains originating from the north Atlantic Ocean. Here, we visualized anti-H3S28P stained embryos from two commercially available antibody sources and unfertilized oocytes to determine the chromosome count of the local Okinawan *O. dioica*.

**Table 1.** Reference to images cited in this study.

Author	Date	Journal	H3S28P source	Figure(s)	Target sample
Spada *et al.*	2005	Journal of Cellular Biochemistry	Thermo Fisher 07-145	3 & 6	Day 3
Schulmeister *et al.*	2007	Chromosome Research	Abcam, ab10543	3 & 5	Male gonad/female coenocyte
Ganot *et al.*	2008	Developmental Biology	Thermo Fisher 07-145	4, 7 & 8	Maturing oocytes
Campsteijn *et al.*	2012	Molecular Biology and Evolution	Abcam, ab10543	1	Hatched larvae
Øvrebø *et al.*	2015	Cell Cycle	Abcam, ab10543	1, 4, 5, 7 & S2A	Maturing oocytes (P3, P4)
Feng & Thompson	2018	Cell Cycle	Abcam, ab10543	1, 2 & 7	P4 ovaries
Olsen *et al.*	2018	BMC Developmental Biology	Abcam, ab10543	5 & Addendum 3	4, 8, 16, 32 cell
Feng *et al.*	2019	Cell Cycle	Abcam ab10543	1, 3, 4, 5 & 6	Hatched larvae

## Methods

### *Oikopleura dioica* culture, staging & preparation of biological material

****Sample preparation.**** Live specimens were collected from Ishikawa Harbor (26 °25'39.3 "N, 127 °49'56.6 "E) by a hand-held plankton net and cultured in the lab ( Masunaga *et al.*, 2020). Mature females were collected prior to spawning, individually washed with filtered autoclaved seawater (FASW) 3 times for 10 minutes and placed in separate 1.5 ml tubes containing 500 µl of FASW. Nearly mature males, full of sperm, were also washed 3 times in FASW. Mature males that successfully made it through the washes intact were placed in 100 µl of fresh FASW and allowed to spawn naturally. As soon as females spawned, each individual clutch of 100–200 eggs was washed three times for 10 minutes by moving eggs along with a pulled capillary micropipette from well to well in a 6-well dish, each containing 5 ml of FASW, and left in a fresh well of 5 ml FASW in the same dish. These were stored at 17 °C and set aside awaiting fertilization. Staged embryos were initiated by gently mixing 10 µl of the spawned male sperm with the awaiting eggs in FASW at 23 °C. Developing embryos were staged and collected by observation under a Leica M165C dissecting microscope. These embryos were quickly dechorionated using 0.1% sodium thioglycolate and 0.01% actinase in FASW for 2–3 minutes, then promptly washed with 2 washes with FASW prior to fixation and staining. Unfertilized eggs were treated similarly with three successive 10-minute washes.

***Histochemical staining.*** Embryos were Giemsa stained as previously described in Shoguchi *et al.*, 2005. Briefly, approximately 20–30 dechorionated embryos were treated with 0.04% colchicine in FASW for 30 minutes and then treated with decreasing amounts of KCl (50 mM and 25 mM) for five minutes each. Fixation was quickly performed with cold methanol:glacial acetic acid (3:1). The fixative was changed three times in the span of 18 hours while at -30 °C. The next morning, the fixed cells were quickly resuspended in 60% Acetic acid and methodically dropped from a height of 7 – 8cm onto a 48°C pre-warmed slide (Matsunami Glass, S2441). The slides were incubated for an additional 2 hours at 48°C; then stained with 6% Gimesa in 67mM sodium phosphate pH 7.0 for 2 hours at room temperature and rinsed with double distilled H _2_O. These were dried for two hours at room temperature, mounted with DPX Mountant (Sigma, 06522) and covered with No.1 35 x 50 mm glass coverslips (Matsunami Glass, C035551).

**Figure 1.**
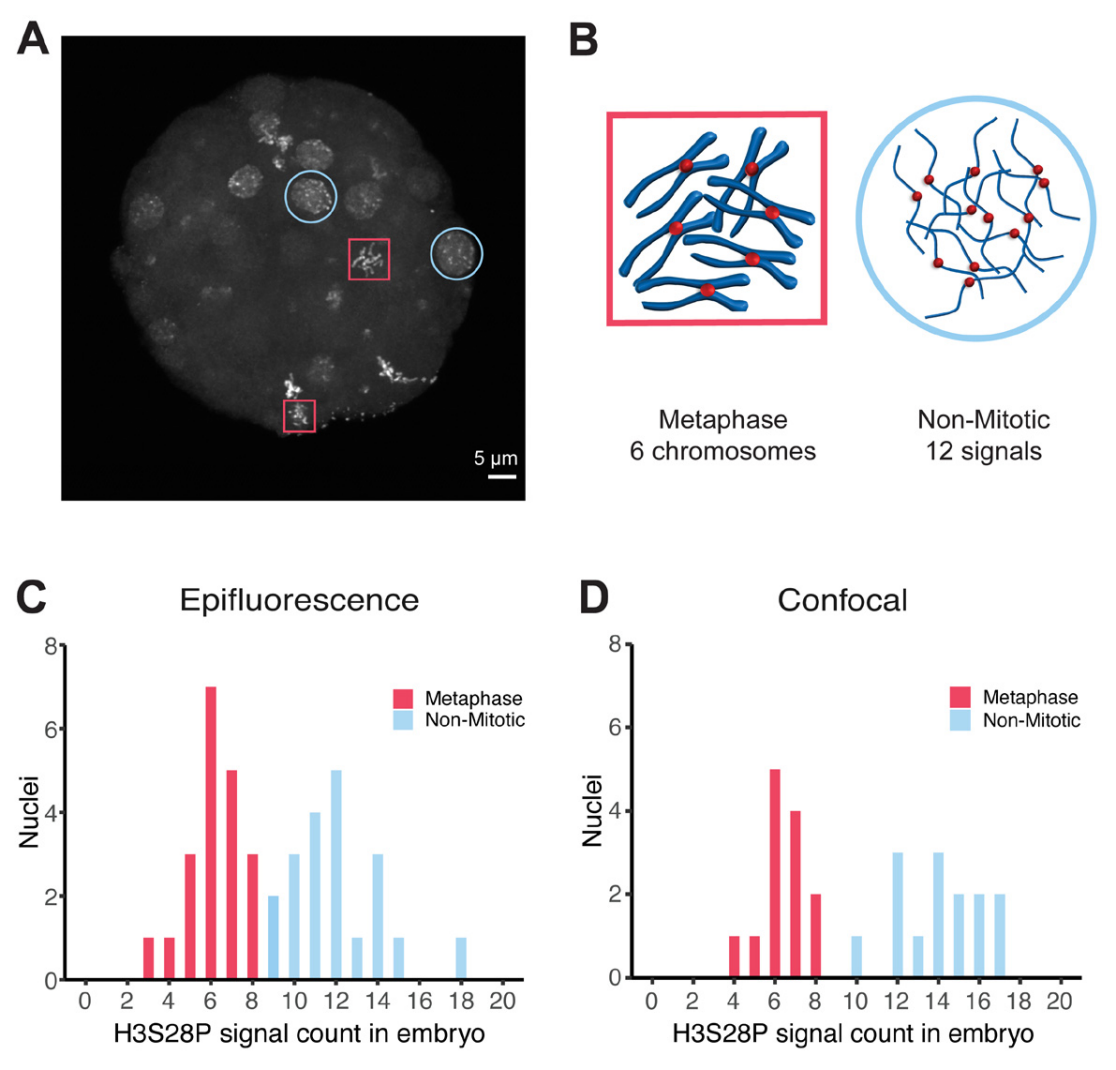
H3S28P signal counts in *O. dioica* embryos. Anti-H3S28P rabbit-derived polyclonal stained 64-cell whole-embryo chromosomal imaging data collected by epifluorescence & confocal microscopy and analyzed by Imaris software SPOT DETECTION tool. **A** Maximum projection of confocal image of an embryo demonstrating the differences in signal localization and count, which was inferred to represent distinct cell cycle phases. (Red box, metaphase; blue circle, non-mitotic; EBI Image Archive S-BIAD21, Experiment D 20191125_01.lsm). **B** Schematic interpretation of signals with respect to chromatin structure during non-mitotic and metaphase cell cycle states. All chromosomes have been drawn with equal lengths for simplicity. **C** Distribution of signal counts within individual cells using epifluorescent (n = 40) and **D** confocal (n = 27) microscopes. The bimodal distribution suggests two distinct populations of cells with different chromosome counts (metaphase, red: epifluorescence n = 20, mean 6.2 , 95% CI 5.6 – 6.8; confocal n = 13, mean 6.4, 95% CI 5.7 – 7.1; non-mitotic, blue: epifluorescence n = 20, mean 12, 95% CI 11.0 – 13.0; confocal, n = 14, mean 14.1, 95% CI 12.9 – 15.3).

**Figure 2.**
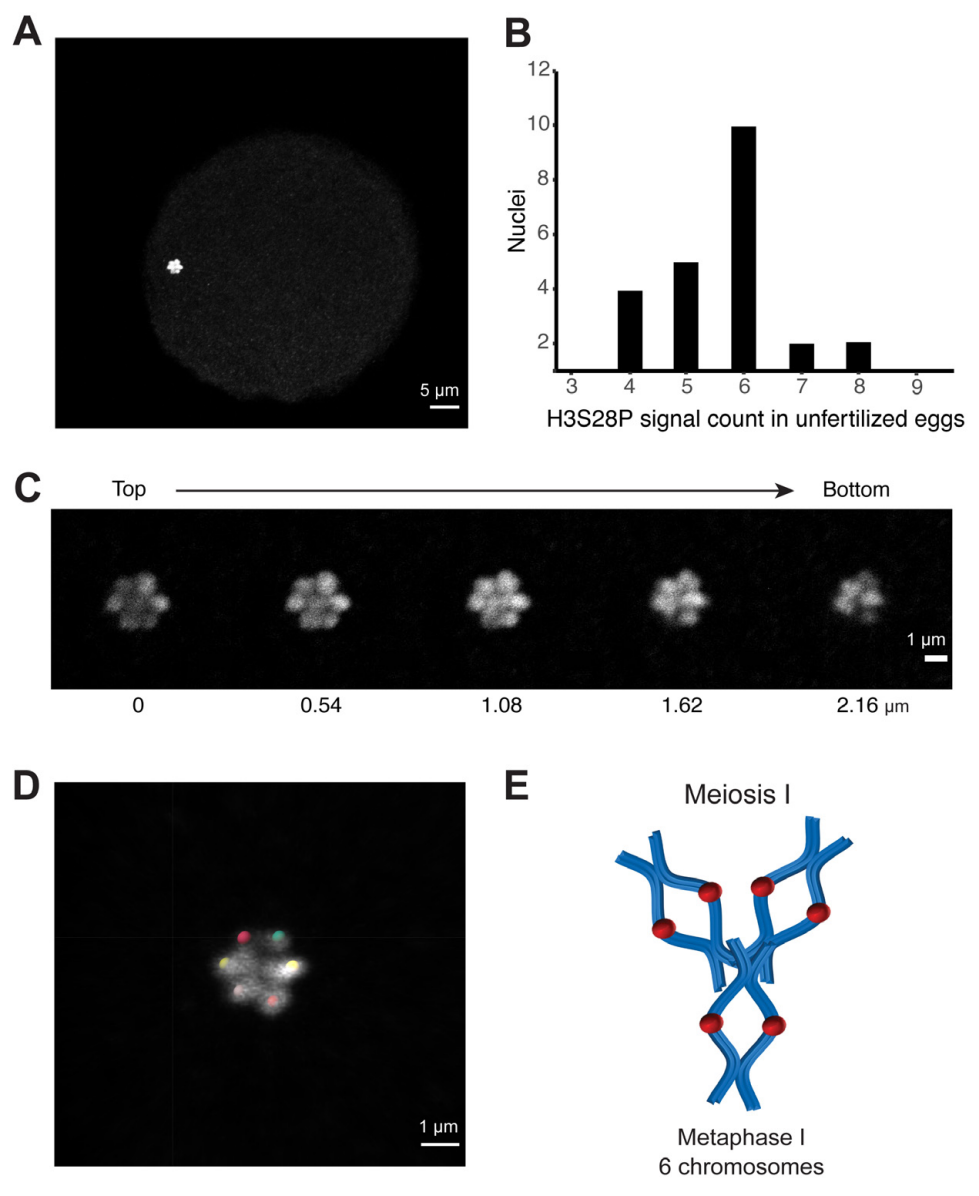
Centromere counts from unfertilized eggs. **A** Maximum signal projection of a representative confocal Z-stack acquisition of anti-H3S28P rat monoclonal stained oocyte used for the count analysis (EBI Image Archive S-BIAD21, Experiment E 20200114_04.lsm). **B** Distribution of signal counts in each rosette-shaped chromatin structure, analyzed by Imaris software SPOT DETECTION tool (n = 23, mean 5.70, 95% CI 5.2 – 6.2). **C** Individual Z-sections from same image acquisition showing the 3D structure of the chromatin. Each plane is 0.54 µm apart. **D** Imaris spot analysis and annotation of signal positions from Z-stack acquisition. **E** Schematic representation of our interpretation that each signal is a centromere from a pair of sister chromatids. Chromosomes have been drawn with equal lengths for simplicity. The positions of centromeric regions cannot be determined as chiasmata(s) are present along the homologous pairs of chromosomes in a highly condensed state.

***Immunostaining.*** Washed eggs, 32 and 64 cell embryos (described above) were immediately fixed in 4% w/v paraformaldehyde, 100 mM MOPS pH 7.5, 0.5 M NaCl, 0.1% triton-X100 at 23 °C overnight ( Campsteijn *et al*., 2012). The samples were then washed for 10 minutes once with PBSTE (PBS supplemented with 1 mM EDTA) and 3 times for 10 min with PBSTEG (PBS supplemented with 1 mM EDTA and 0.1 M glycine). The samples were blocked using PBSTE supplemented with 3% bovine serum albumin at 4 °C overnight. Rabbit polyclonal ( Figure 1; Thermo Fisher Scientific Cat# 720099, RRID:AB_2532807) or rat monoclonal ( Figure 2; Abcam Cat# ab10543, RRID:AB_2295065) primaries directed against H3S28P were diluted 1:100 in PBSTE 3% BSA and incubated at 4 °C for 3 days. The next morning, these were washed in PBSTE for 10 minutes 3 times and incubated with anti-rabbit (Thermo Fisher Scientific Cat# A-11034, RRID:AB_2576217) or anti-rat (Molecular Probes Cat# A-11006, RRID:AB_141373) Alexa488 conjugated secondary antibodies diluted 1:500 with PBSTE 3% BSA at 4 °C overnight. The following morning, samples were washed 3 times for 10 min with PBSTE. The samples were mounted on cleaned glass slides (Matsunami Glass, S2441) with fluorescence preserving mounting medium (ProLong. Fluoromount G Mounting Medium, RRID:SCR_015961) covered with No.1 35 x 50 mm glass coverslips (Matsunami Glass, C035551) and sealed with nail polish.

### Image acquisition

Both a Nikon Ni-E epifluorescent and a Zeiss LSM 510 Meta confocal microscopes were used to acquire Z-stack images of eggs and embryos. Brightfield images were obtained using a 20x/0.75 CFI Plan Apo λ objective (Nikon, MRD00205) for histochemical staining. Epifluorescent immunofluorescent images were obtained with both 20x/0.75 and 40x/0.95 CFI Plan Apo λ air objectives (Nikon, MRD00405); each sample acquisition was Z-stacked with each plane set at an interval of 1 µm. Confocal images were acquired using a 40x/0.75 EC Plan-Neofluar M27 (Zeiss, 420360-9900-000) and 63x/1.4 Plan-Apochromat M27 oil immersion (Zeiss, 420782-9900-79) objectives; each sample acquisition was Z-stacked, line averaged twice with each plane set at an interval of 0.6 and 0.27 µm, respectively.

### Image processing and analysis

Images acquired from a Nikon Ni-E epifluorescent were deconvoluted with Nikon Elements-AR v5.0 software. Images for both epifluorescent and confocal acquisitions were analyzed using Imaris software SPOT DETECTION tool (Imaris, RRID:SCR_007370) for embryos and unfertilized eggs, parameters set at 0.5 and 0.43 µm spot detection size, respectively, and software preset to QUALITY auto signal threshold for each individual cell within a sample. Alternatively, ImageJ v1.51 3D Objects Counter may be employed to count signals. Epifluorescent and confocal acquisitions of embryos and their subsequent analysis were performed independently by different researchers to exclude bias.

### Statistical analysis

Confidence intervals were calculated with Prism 8 (GraphPad) and histograms plotted with R (v3.6.3).

## Results

We initially attempted to visualize chromosomes using Giemsa staining on developing embryos. The spreads from 32- and 64-cell developmental stages, gave results with counts ranging between 11–27 stains per cell (BioImage Archive, S-BIAD21, Experiment A). Although cell-spreads were confined as a result of incomplete dechorionation with the enzymatic dissociation cocktail, we were still able to assign chromosomes to individual cells. Disappointingly, chromosome counts were unreliable due to the observed variability.

Consequently, we performed immunostaining of similarly staged embryos using a H3S28P-specific primary antibody and a secondary antibody conjugated to Alexa488 directed against the primary antibody. Signal-based thresholding was employed to determine the number of distinct 515 nm emission signals present in images acquired with epifluorescent and laser confocal microscopes (BioImage Archive, S-BIAD21, Experiment B & D). The data was analyzed using the Imaris SPOT DETECTION tool (Oxford Instruments).

Cells were manually classified into two types depending on the staining pattern visible in the nucleus: (i) those with intense clusters of signals in the center, considered to be in metaphase and (ii) those containing evenly distributed, clearly separated spots within a faint background of signal defining a region encompassed by the nuclear envelope, interpreted as non-mitotic ( Figure 1A and 1B, blue circles; Figure 1A and 1B, red squares). Counts from these two classes of nuclei fall into separate distributions ( Figure 1C and 1D), with both epifluorescence and confocal acquisitions in agreement with each other. We interpreted the nuclei with an average of six large, clustered signals as centromeric regions in metaphase ( Figure 1B), however, we cannot explain the cell cycle state of those containing the average of 12 spatially distinct punctate signals.

To rule out polyploidy, which occurs in *O. dioica* somatic cells that give rise to the mucosal house ( Ganot & Thompson, 2002), we also analyzed oocytes in metaphase I before fertilization ( Schulmeister *et al.*, 2007). We identified confined groupings of signals in unfertilized eggs ( Figure 2A; BioImage Archive, S-BIAD21, Experiment E) and analyzed confocal images using the Imaris SPOT DETECTION tool to determine H3S28P signal counts ( Figure 2B). Counts from the compact rosette-shaped chromatin structure averaged near 6. Visual inspection of individual Z-sections ( Figure 2C) confirms the Imaris count analysis and annotation ( Figure 2D). We interpreted each spot as representing a centromere from paired chromatids forming a synapsis in unfertilized eggs ( Figure 2E).

## Discussion

Our initial attempts at karyotyping by traditional Giemsa-staining gave us wildly varying counts which we unable to overcome with or without mitotic arrest. Giemsa-staining has been applied successfully to other organisms with small chromosomes such as the tunicate *Ciona intestinalis* ( Shoguchi *et al.*, 2005). The difference in outcome might be explained by the higher AT content of those genomes compared with *O. dioica*, since Giemsa preferentially stains AT-rich sequences. Although we do rule out Giemsa-staining as an effective method for studying *O. dioica* chromosomes, in our hands, immunostaining yielded more consistent results.

Most karyotyping studies display a representative image to support the conclusion; however, given the variability in signal counts between nuclei, we decided to take a statistical approach that quantifies the uncertainty in the estimated chromosome count. Despite testing many different image acquisition settings, we were unable to eliminate the variability; we believe there are several possible reasons that explain the variance. (i) We applied uniform signal thresholds to all cells, so any spots below the threshold would have been missed. (ii) Spots displayed non-uniform signals, and individual centromeres may have occasionally contributed multiple counts. (iii) The H3S28P signal is not always confined to centromeres, and so may have caused multiple counts (see below). (iv) Finally, the three-dimensional rosette structures in oocytes might not have always been captured reliably in the focal plane. It is worth noting that for *O. dioica*, immunostaining showed much smaller variabilities than Giemsa-staining.

An important consideration is what the H3S28P signal represents. It has been used to visualize centromeric regions in *O. dioica* ( Table 1), but the signal is not confined to the centromere and its localization depends on the cellular state ( Figure 1; Hake *et al.*, 2005; Feng & Thompson, 2018). However, we are confident that the signals seen in Figure 1 labelled as metaphase and Figure 2 represent centromeres and their associated chromosome. Further, DNA-staining images of mature oocyte have previously been interpreted as chromosomes condensed in a structure resembling the Greek character ∏ ( Ganot *et al.*, 2007; Ganot *et al.*, 2007b; Ganot *et al.*, 2008). Since we did not perform DNA stains, our interpretation of the H3S28P signal in the oocyte does not preclude the previously reported ∏-structure. Additionally, the positions and numbers of crossovers between homologous pairs are unresolvable in this highly condensed state and the signal positions are not definitive of centromeric regions.

Currently, the nucleotide sequence of the centromeric region is unknown for *O. dioica*, although chromatin immunoprecipitation with a H3S28P antibody followed by long-read sequencing might be able to provide this information. However, our whole embryo staining data ( Figure 1) and the previous literature ( Table 1) show that the H3S28P antibody produces non-centromeric signals which may confound such analysis. Thus, alternative targets such as other centromeric histone 3 variants ( Moosmann *et al.*, 2011) might be preferable. Knowledge of centromeric sequences would also open the possibility of confirming these results with fluorescence *in situ* hybridization.

Despite the variations in signal counts between nuclei, a haploid chromosome count of three provides the most parsimonious explanation of the collected data and is consistent with previously published genome sequence assemblies ( Denoeud *et al.*, 2010). In summary, we conclude that the Okinawan *Oikopleura dioica* genome consists of three pairs of chromosomes in diploid cells. We believe that the images may be useful for examining cell cycle specific changes to chromosome structure and encourage the reuse and reanalysis of our data located in the EBI BioImage Archive ( Ellenberg *et al.*, 2018).

## Data availability

### Underlying data

Image acquisitions: Image data are available from the BioImage Archive Accession number S-BIAD21 ( https://www.ebi.ac.uk/biostudies/studies/S-BIAD21)
