# Supplementary material for: H3S28P Antibody Staining of Okinawan  Oikopleura dioica Suggests the Presence of Three Chromosomes
Source: F1000Res. 2021 Mar 1;9:780. Originally published 2020 Jul 28. [Version 2] doi: 10.12688/f1000research.25019.2 (PMC7941098; doi:10.12688/f1000research.25019.2)
Supplement: Peer review history

# Peer review: Reviewer response for version 2

**DOI:** 10.5256/f1000research.54241.r80403

**Published:** 2021-03-08

**Authors:** Shigeki Fujiwara

**Affiliations:** 1 Department of Chemistry and Biotechnology, Faculty of Science and Technology, Kochi University, Kochi, Japan

All of the concerns I had raised were carefully addressed. I feel that the revised version of the manuscript became much clearer and convincing.

Is the work clearly and accurately presented and does it cite the current literature?

Partly

If applicable, is the statistical analysis and its interpretation appropriate?

I cannot comment. A qualified statistician is required.

Are all the source data underlying the results available to ensure full reproducibility?

Yes

Is the study design appropriate and is the work technically sound?

Yes

Are the conclusions drawn adequately supported by the results?

Yes

Are sufficient details of methods and analysis provided to allow replication by others?

Yes

Reviewer Expertise:

Tunicate embryogenesis, asexual reproduction, and evolutionary developmental biology.  Transcriptional regulation.

I confirm that I have read this submission and believe that I have an appropriate level of expertise to confirm that it is of an acceptable scientific standard.

# Peer review: Reviewer response for version 2

**DOI:** 10.5256/f1000research.54241.r80404

**Published:** 2021-03-02

**Authors:** Haiyang Feng

**Affiliations:** 1 Sars International Centre for Marine Molecular Biology, University of Bergen, Bergen, Norway

Overall, I'm satisfied with the revision. As to the H3S28p signals in embryonic cells not at metaphase, it seems that these cells are at early prophase, since interphase during 32C to 64C stages should be very short. I would interpret the H3S28p dots of 8 to 18 early on at prophase are on chromosomes, but not necessarily on centromeres.

Is the work clearly and accurately presented and does it cite the current literature?

Yes

If applicable, is the statistical analysis and its interpretation appropriate?

I cannot comment. A qualified statistician is required.

Are all the source data underlying the results available to ensure full reproducibility?

Yes

Is the study design appropriate and is the work technically sound?

Yes

Are the conclusions drawn adequately supported by the results?

Yes

Are sufficient details of methods and analysis provided to allow replication by others?

Yes

Reviewer Expertise:

cell cycle, oogenesis

I confirm that I have read this submission and believe that I have an appropriate level of expertise to confirm that it is of an acceptable scientific standard.

# Peer review: Reviewer response for version 1

**DOI:** 10.5256/f1000research.27598.r68262

**Published:** 2020-08-12

**Authors:** Shigeki Fujiwara

**Affiliations:** 1 Department of Chemistry and Biotechnology, Faculty of Science and Technology, Kochi University, Kochi, Japan

This manuscript describes a new method for karyotyping using the antibody raised against Ser28-phosphorylated Histone H3 (H3S28P). Using this method, the authors obtained the results suggesting that Okinawan *Oikopleura dioica *somatic cells contain three sets of chromosomes. Specific detection of *O. dioica*’s phosphorylated H3 by the antibody has been proven in other papers, shown in Table 1. The data presented in this article are therefore reliable, and the conclusion seems appropriate. However, after I read to the end of the article, I did not really understand what the main aim and novelty of this article were. Which is the main aim, development of a new karyotyping method or determination of the number of chromosomes in diploid *O. dioica* somatic cells? Although the article type is “BRIEF REPORT”, clearer statements and more detailed explanations are required. I hope that the following comments are useful for the authors. All of my comments are for presentation and description.

**Major concerns:**

1. The Introduction section starts with the history of karyotyping. This implies that the development of a new karyotyping technique appears to be the main aim of this study. The authors intend to argue the advantage of the karyotyping method using H3S28P-specific antibody. However, the authors observed fairly large variation in the number of H3S28P signals (number of centromeres). Shoguchi *et al.* (2005) (cited in this article) clearly showed 14 pairs of chromosomes of the *Ciona intestinalis *genome by means of Giemsa staining and FISH. While the size of the genome in *O. dioica* is a half of that in *C. intestinalis*, the number of chromosomes in *O. dioica* is about one-fifth of that in *C. intestinalis*. Therefore, readers may feel that the average size of the *O. dioica* chromosomes is large enough to be examined by the standard methods. If the development of the new method is really the main aim of this study, I would like the authors to describe merits of this new method in further detail. Without sufficiently convincing explanations, the authors’ method appears to be a less sophisticated alternative to the standard karyotyping methods. Particularly, discussion is required for the observation of seven or eight signals within a single nucleus. It will help if the authors explain why the standard methods are not applicable to *O. dioica*.
2. If the authors’ main aim is to determine the number of chromosomes in Okinawan *O. dioica*, they should explain more about particularity of this species. Is there a hypothesis that Pacific and Atlantic *O. dioica* are different species?  If not, is there the possibility that different populations (Pacific and Atlantic) have different numbers of chromosomes within the same species? The number of chromosomes is highly variable even between closely related species. However, to my knowledge, the number of chromosomes is essentially invariant within a species. Uncommon exceptions are chromosome reorganization in Ascaris embryos and Paramecium macronuclei. Although the authors discuss the discrepancy in the number of the *O. dioica *chromosomes (n = 3, or n = 8), I felt that the argument has already been settled (on n = 3) by the extensive genome sequencing (Denoeud *et al.*, 2010). If the authors want to insist that the number of chromosomes in Pacific *O. dioica* may not be three, more detailed biological information (rationale) is necessary.

 

**Minor points:**

1. In Table 1, “Ganot *et al.*” should be “Ganot & Thompson”. Similarly, “Feng *et al. *(2018)” should be “Feng & Thompson (2018)”.
2. I guess that “ddH2O” (page 4 line 17) is double-distilled H _2_O. Anyway, “ddH2O” is a laboratory-specific jargon. Similarly, I guess that “ON” (page 4 line 23) means “overnight”?  These abbreviations cannot be recommended to be used in articles.
3. Since the authors have knowledge that some somatic cells are polyploid in *O. dioica.* Therefore, they had better clearly state that the cells shown in Figure 1 are not the case. Although the authors state that 32~64-cell embryos were used for Giemsa staining, they did not tell the developmental stages they used for the antibody staining (in the second paragraph of the Results section). Are they also the early embryos? And do they consist exclusively of diploid cells?

Is the work clearly and accurately presented and does it cite the current literature?

Partly

If applicable, is the statistical analysis and its interpretation appropriate?

I cannot comment. A qualified statistician is required.

Are all the source data underlying the results available to ensure full reproducibility?

Yes

Is the study design appropriate and is the work technically sound?

Yes

Are the conclusions drawn adequately supported by the results?

Yes

Are sufficient details of methods and analysis provided to allow replication by others?

Yes

Reviewer Expertise:

Tunicate embryogenesis, asexual reproduction, and evolutionary developmental biology.  Transcriptional regulation.

I confirm that I have read this submission and believe that I have an appropriate level of expertise to confirm that it is of an acceptable scientific standard, however I have significant reservations, as outlined above.

# Response

**Published:** 2021-01-29

**Authors:** Andrew W Liu

**Affiliations:** Okinawa Institute of Science and Technology, Japan

We thank Dr Fujiwara’s helpful feedback and critique on our manuscript. We have done our best to address all the concerns and minor points he has brought to our attention, which are listed below.

**Reviewer 2 synopsis**

**Reviewer comment**

 This manuscript describes a new method for karyotyping using the antibody raised against Ser28-phosphorylated Histone H3 (H3S28P). Using this method, the authors obtained the results suggesting that Okinawan Oikopleura dioica somatic cells contain three sets of chromosomes. Specific detection of O. dioica’s phosphorylated H3 by the antibody has been proven in other papers, shown in Table 1. The data presented in this article are therefore reliable, and the conclusion seems appropriate. However, after I read to the end of the article, I did not really understand what the main aim and novelty of this article were. Which is the main aim, development of a new karyotyping method or determination of the number of chromosomes in diploid O. dioica somatic cells? 

 Although the article type is “BRIEF REPORT”, clearer statements and more detailed explanations are required. I hope that the following comments are useful for the authors. All of my comments are for presentation and description.

**Author response** 

 We thank the referee for the feedback, which have helped improve the clarity and quality of the manuscript. To clarify, the aim of this paper is to determine the number of chromosomes for the Okinawan *O. dioica *genome. We have detailed the reasons for this in our response to Reviewer Comment 1.1 above. 

**Manuscript changes** 

 We clarified the main aim of the paper and strengthened the justification for this in the Abstract and Introduction (please see authors response to Reviewer Comment 1.1).

 _____________________

**Major concerns**

**Reviewer 2 comment** **1.1** **–** **Clarification of study aim**

**Reviewer comment**

 The Introduction section starts with the history of karyotyping. This implies that the development of a new karyotyping technique appears to be the main aim of this study. 

**Author response** 

 We thank the referee for this comment. We have now substantially revised the Introduction to clarify that the main aim of the study is to determine the chromosome count. We have retained the description of the histochemical and immunostaining methods as two contrasting approaches, in order to explain why we chose the latter approach here; however, we hope that it is now clear that we are not implying the publication of a new karyotyping technique.

**Manuscript changes** 

 1. Abstract. “ *Oikopleura dioica* is a ubiquitous marine zooplankton of biological interest owing to features that include dioecious reproduction, a short life cycle, conserved chordate body plan, and a compact genome. It is an important tunicate model for evolutionary and developmental research, as well as investigations into marine ecosystems. The genome of north Atlantic *O. dioica *comprises three chromosomes. However, comparisons with the genomes of *O. dioica *sampled frommainland and southern Japan revealed extensive sequence differences. Moreover, historical studies have reported widely varying chromosome counts. We recently initiated a project to study the genomes of *O. dioica* individuals collected from the coastline of the Ryukyu (Okinawa) Islands in southern Japan. Given the potentially large extent of genomic diversity, we employed karyological techniques to count individual animals’ chromosomes *in situ* using centromere-specific antibodies directed against H3S28P, a prophase-metaphase cell cycle-specific marker of histone H3. Epifluorescence and confocal images were obtained of embryos and oocytes stained with two commercial anti-H3S28P antibodies (Abcam ab10543 and Thermo Fisher 07-145). The data lead us to conclude that diploid cells from Okinawan *O. dioica* contain three pairs of chromosomes, in line with the north Atlantic populations. The finding facilitates the telomere-to-telomere assembly of Okinawan *O. dioica *genome sequences and give insight into the genomic diversity of *O. dioica* from different geographical locations. The data deposited in the EBI BioImage Archive provide representative images of the antibodies’ staining properties for use in epifluorescent and confocal based fluorescent microscopy.”

 2. Paragraph 1 (Introduction). “… Given the large sequence and synteny differences between the assembled *O. dioica *genomes, as well as the discrepancies among previous studies, we wished to assess the karyotype for the local Okinawan *O. dioica *population.”

 3. Paragraph 2 (Introduction). “However, we were unable to resolve individual *O. dioica *chromosomes by this method [Giemsa staining]...”

 4. Paragraph 3 (Introduction). “As an alternative approach, we decided to immunostain the centromere as a means of quantifying the numbers of chromosomes… Here, we visualized anti-H3S28P stained embryos from two commercially available antibody sources and unfertilized oocytes to determine the chromosome count of the local Okinawan *O. dioica*.”

 _____________________

**Reviewer 2 comment** **1.2** **–** **Variability of data**

**Reviewer comment**

 The authors intend to argue the advantage of the karyotyping method using H3S28P-specific antibody. However, the authors observed fairly large variation in the number of H3S28P signals (number of centromeres).

 Particularly, discussion is required for the observation of seven or eight signals within a single nucleus.

**Author response** 

 We thank the referee for this comment.

 1. Despite the apparent certainty in chromosome numbers, variability in signal counts does not appear to be unusual. For instance, Fenaux and Colombera noted (1973) reported “In another five anaphase plates, presumably because of chromosome losses during the squashing, a lower number was found.” However, most karyological papers generally present a very small number of representative images; therefore, we cannot comment on whether the variation we observe is unusually large compared with other studies. It is worth noting that in our hands, Giemsa-staining yielded even larger variability than immunostaining in our hands.

 2. It is because of this variability that we decided to use a statistical approach: calculating  confidence intervals allows us to quantify the uncertainty in the conclusions that we draw from each set of experiments, fully accounting for the variability.

 3. We have added a discussion of the possible sources of variation in the number of H3S28P signals. Specifically, we believe that nuclei containing 7-8 counts, arise from non-uniform spots being split into multiple counts; for individual nuclei, this could be resolved by adjusting the signal threshold, but this is not possible if a uniform threshold is applied across all nuclei.

 4. From a statistical perspective, the intervals are all narrow and centre on a mean of 6 across three different experimental set ups  (Figure 1: mean 6.2, 95% CI 5.6 – 6, mean 6.4, 95% CI 5.7 – 7.1; Figure 2: mean 5.70, 95% CI 5.2 – 6.2). From a biological perspective, the observation is consistent with our genome sequence assembly. Together, these give us reasonable confidence that we have reached the correct conclusion that there are three chromosomes.

**Manuscript changes** 

 Paragraph 15 (Discussion). “Most karyotyping studies display a representative image to support the conclusion; however, given the variability in signal counts between nuclei, we decided to take a statistical approach that quantifies the uncertainty in the estimated chromosome count. Despite testing many different image acquisition settings, we were unable to eliminate the variability; we believe there are several possible reasons that explain them. (i) We applied uniform signal thresholds to all cells, so any spots below the threshold would have been missed. (ii) Spots displayed non-uniform signals, and individual centromeres may have occasionally contributed multiple counts. (iii) The H3S28P signal is not always confined to centromeres, and so may have caused multiple counts (see below). (iv) Finally, the three-dimensional rosette structures in oocytes might not have always been captured reliably in the focal plane. It is worth noting that for *O. dioica*, immunostaining showed much smaller variabilities than Giemsa-staining.”

 _____________________

**Reviewer 2 comment 1.3** **–** **Use of immunostaining over histochemical methods**

**Reviewer comment**

 Shoguchi et al. (2005) (cited in this article) clearly showed 14 pairs of chromosomes of the Ciona intestinalis genome by means of Giemsa staining and FISH. While the size of the genome in O. dioica is a half of that in C. intestinalis, the number of chromosomes in O. dioica is about one-fifth of that in C. Intestinalis. Therefore, readers may feel that the average size of the O. dioica chromosomes is large enough to be examined by the standard methods. If the development of the new method is really the main aim of this study, I would like the authors to describe merits of this new method in further detail. Without sufficiently convincing explanations, the authors’ method appears to be a less sophisticated alternative to the standard karyotyping methods.

 It will help if the authors explain why the standard methods are not applicable to O. dioica. 

**Author response** 

 We thank the referee for this comment. We were equally frustrated by the difficulties in performing Giemsa staining, which gave even larger variations in signal counts. Anecdotally, this appears to be a similar experience in other laboratories studying *O. dioica*. FISH is an attractive future possibility for further validation of the immunostaining and genome assembly results.

**Manuscript changes**

 Please also see authors response to Reviewer 1 comment 3.1 ****– *Interpretation of H3S28P signal* locations above.

 1. Paragraph 14 (Discussion). “Our initial attempts at karyotyping by traditional Giemsa staining gave us wildly varying counts which we unable to overcome with or without mitotic arrest. Giemsa-staining has been applied successfully to other organisms with small chromosomes such as the tunicate *Ciona intestinalis* (Shoguchi et al., 2005). The difference in outcome might be explained by the higher AT content of those genomes compared with *O. dioica*, since Giema preferentially stains AT-rich sequences. Although we do rule out Giema-staining as an effective method for studying *O. dioica* chromosomes, in our hands, immunostaining yielded more consistent results.”

 2. Paragraph 17 (Discussion). “Currently, the nucleotide sequence of the centromeric region is unknown for *O. dioica*, although chromatin immunoprecipitation with a H3S28P antibody followed by long-read sequencing might be able to provide this information. However, our whole embryo staining data (Figure 1) and the previous literature (Table 1) show that the H3S28P antibody produces non-centromeric signals which may confound such analysis. Thus, alternative targets such as other centromeric histone 3 variants (Moosmann *et al.*, 2011) might be preferable. Knowledge of centromeric sequences would also open the possibility of confirming these results with fluorescence *in situ *hybridization.”

 _____________________

**Reviewer 2 comment** **2.1** **–** **Rationale of study**

**Reviewer comment**

 If the authors’ main aim is to determine the number of chromosomes in Okinawan O. dioica, they should explain more about particularity of this species. Is there a hypothesis that Pacific and Atlantic O. dioica are different species? 

 If not, is there the possibility that different populations (Pacific and Atlantic) have different numbers of chromosomes within the same species?  The number of chromosomes is highly variable even between closely related species. However, to my knowledge, the number of chromosomes is essentially invariant within a species. Uncommon exceptions are chromosome reorganization in Ascaris embryos and Paramecium macronuclei. Although the authors discuss the discrepancy in the number of the O. dioica chromosomes (n = 3, or n = 8), I felt that the argument has already been settled (on n = 3) by the extensive genome sequencing (Denoeud et al., 2010). If the authors want to insist that the number of chromosomes in Pacific O. dioica may not be three, more detailed biological information (rationale) is necessary.

**Author response** 

 We thank the referee for this comment. Please also see authors response to Reviewer 1 Comment 1.1.

 Briefly, we observe large genome sequence variations between north Atlantic, mainland Japanese and Okinawan *O. dioica* samples, both at nucleotide level and kilo-megabase scale. This is why we decided to check the number of chromosomes in the Okinawan *O. dioica*. We feel it’s too early to conclude whether they represent distinct species.

 Regarding the earlier literature, since Colombera and Fenaux (1973) reported 8 chromosomes, it is possible that they examined a different species of Oikopleura.

**Manuscript changes** 

 Please see authors response to Reviewer 1 Comment 1.1

 _____________________

**Minor points**

**Reviewer 2 minor point** **1**

**Reviewer comment**

 In Table 1, “Ganot et al.” should be “Ganot & Thompson”. Similarly, “Feng et al. (2018)” should be “Feng & Thompson (2018)”. 

**Author response** 

 We have made the changes to the citations in the manuscript as suggested.

**Manuscript changes** 

 1. Feng et al. (2018) was changed to “Feng & Thompson”.

 2. Ganot *et al. *(2008) was left unchanged as it refers to Ganot P, Schulmeister A, Thompson EM, (2008).

 ___________________

**Reviewer 2 minor point 2**

**Reviewer comment**

 I guess that “ddH2O” (page 4 line 17) is double-distilled H _2_O. Anyway, “ddH2O” is a laboratory-specific jargon. Similarly, I guess that “ON” (page 4 line 23) means “overnight”?  These abbreviations cannot be recommended to be used in articles. 

**Author response** 

 We have replaced jargon and abbreviations with full terminology in the methods sections.

**Manuscript changes** 

 Changes made in paragraph 5 and 6.

 _____________________ 

**Reviewer 2 minor point 3**

**Reviewer comment**

 Since the authors have knowledge that some somatic cells are polyploid in O. dioica. Therefore, they had better clearly state that the cells shown in Figure 1 are not the case. Although the authors state that 32~64-cell embryos were used for Giemsa staining, they did not tell the developmental stages they used for the antibody staining (in the second paragraph of the Results section). Are they also the early embryos? And do they consist exclusively of diploid cells? 

**Author response** 

 We updated the methods section to indicate the developmental stage of the stained embryos (“ *32 and 64-cell embryos*”) and underlined that the same stage was used for Giemsa and antibody staining by adding the words “similarly staged embryos” in the first paragraph of the results section. It is our understanding that the polyploid cells outlined in Ganot & Thompson, 2002 which are responsible for the extrusion of the mucosal house are present in the later stages of development.

**Manuscript changes**

 1. ****Paragraph 6. “Washed eggs, 32 and 64 cell embryos (described above) were immediately fixed…”

 2. Paragraph 11. “Consequently, we performed immunostaining of similarly staged embryos…”

 3. Paragraph 13. “To rule out polyploidy, which occurs in *O. dioica *somatic cells that give rise to the mucosal house (Ganot & Thompson, 2002), we also analyzed oocytes in metaphase I before fertilization”

# Peer review: Reviewer response for version 1

**DOI:** 10.5256/f1000research.27598.r68260

**Published:** 2020-08-05

**Authors:** Haiyang Feng

**Affiliations:** 1 Sars International Centre for Marine Molecular Biology, University of Bergen, Bergen, Norway

It’s interesting to know, though not surprising, that Japanese *O. dioica* has the same number of centromeres and chromosomes as that in Norwegian species. This piece of work can boost broad interests in using *O. dioica* as a new model in epigenetics and cell cycle studies. However, some results are a bit confusing to me and may be misinterpreted.

 In Fig 1, centromere counts at prophase are 12, and at metaphase are 6, which are inconsistent. H3S28p signals locate at inner centromeric regions, flanked by CenpA signals that mark kinetochores at metaphase in embryonic mitosis in Norwegian *O. dioica*. The counts of H3S28p signals should be the same at prophase and metaphase, which are 6. In addition, centromere is a piece of DNA sequence that holds a pair of sister chromatids in mitotic phase before they separate at anaphase. We can say that a chromosome has one centromere and a pair of sister chromatids at prophase. Thus, the schema representing prophase in Fig 1B should be a pair of sister chromatids is linked by one red dot at centromere.

 H3S28p signals in female meiosis of Norwegian *O. dioica* are a bit different from those in mitosis. It localizes on entire chromosomes in prophase, moves towards centromeric regions during prometaphase, and is enriched at centromeric regions (or accurately speaking, midline of a bivalent) at metaphase I. Since chromosomes are more condensed in meiosis, and the midline of a bivalent should be crossover site between homologous chromosomes, we don’t know how far away it is between centromere and crossover in meiotic chromosomes of *O. dioica*, and how many crossovers a bivalent has. I would say centromeric region of H3S28p signals in female meiosis with caution. Actually, H3S28p shows several spots (more than 6) during prometaphase I, as can be seen Fig S4 in Feng and Thompson, 2018 cell cycle. The stages of meiosis depend on when the oocytes are collected. Just after spawning, the oocytes are before prometaphase I. Within 10 to 15 min after spawning, it is prometaphase I. Later, it should be at metaphase I. The timing of sampling is not indicated, which makes it even harder to interpret the data. But again, it should one red dot between a pair of sister chromatids in Fig 2E.

Is the work clearly and accurately presented and does it cite the current literature?

Yes

If applicable, is the statistical analysis and its interpretation appropriate?

I cannot comment. A qualified statistician is required.

Are all the source data underlying the results available to ensure full reproducibility?

Yes

Is the study design appropriate and is the work technically sound?

Yes

Are the conclusions drawn adequately supported by the results?

Yes

Are sufficient details of methods and analysis provided to allow replication by others?

Yes

Reviewer Expertise:

cell cycle, oogenesis

I confirm that I have read this submission and believe that I have an appropriate level of expertise to confirm that it is of an acceptable scientific standard, however I have significant reservations, as outlined above.

# Response

**Published:** 2021-01-29

**Authors:** Andrew W Liu

**Affiliations:** Okinawa Institute of Science and Technology, Japan

We thank Dr Feng’s helpful feedback on our manuscript. We have done our best to address all the comments, which are listed below.

 An aspect of the data that puzzles us is the evenly distributed, clearly separated H3S28P signal observed in non-mitotic cells, which we incorrectly referred to as prophase in the previous version of the manuscript (please see responses 2.1 and 3.1). We have consulted cell cycle experts who were unable to explain the results. Given Dr Feng’s extensive experience with H3S28P staining, we would like to ask if these patterns have been observed in his laboratory? We’d be grateful to organize a videoconference with Dr Feng to discuss the data archived at the EBI BioArchive to share our observations in detail.

 _____________________

**Reviewer 1 comment 1.1 –** **Comparison of chromosome numbers between Japanese and Norwegian *O. dioica***

**Reviewer comment**

 It’s interesting to know, though not surprising, that Japanese O. dioica has the same number of centromeres and chromosomes as that in Norwegian species.

**Author response** 

 We thank the reviewer for this comment.

 1. After submission of this manuscript to F1000Research, two additional *O. dioica *genomes were published for (i) samples acquired in mainland Japan and (i) an individual from the Okinawa coastline. Preliminary comparison of the three *O. dioica*  genomes have revealed very divergent sequences at single nucleotide and kilo/megabase scales (unpublished results). Given that mainland and Okinawan *O. dioica *are both “Japanese”, we avoid the term “Japanese *O. dioica*” in the present manuscript.

 2. Historical studies reported between 3 and 8 chromosomes for *O. dioica*.

 3. For these reasons, it was not obvious to us that the Okinawan *O. dioica* would have the same number of chromosomes as the Norwegian and mainland Japanese *O. dioica.*

 4. Therefore, we wished to confirm independently the chromosome number of the Okinawan O. dioica.

**Manuscript changes** 

 We rewrote the Abstract and Introduction to strengthen the justification for this study.

 Abstract. *Oikopleura dioica* is a ubiquitous marine zooplankton of biological interest owing to features that include dioecious reproduction, a short life cycle, conserved chordate body plan, and a compact genome. It is an important tunicate model for evolutionary and developmental research, as well as investigations into marine ecosystems. The genome of north Atlantic *O. dioica *comprises three chromosomes. However, comparisons with the genomes of *O. dioica *sampled from **mainland and southern Japan revealed extensive sequence differences. Moreover, historical studies have reported widely varying chromosome counts. We recently initiated a project to study the genomes of *O. dioica*individuals collected from the coastline of the Ryukyu (Okinawa) Islands in southern Japan. Given the potentially large extent of genomic diversity, we employed karyological techniques to count individual animals’ chromosomes *in situ* using centromere-specific antibodies directed against H3S28P, a prophase-metaphase cell cycle-specific marker of histone H3. Epifluorescence and confocal images were obtained of embryos and oocytes stained with two commercial anti-H3S28P antibodies (Abcam ab10543 and Thermo Fisher 07-145). The data lead us to conclude that diploid cells from Okinawan *O. dioica*contain three pairs of chromosomes, in line with the north Atlantic populations. The finding facilitates the telomere-to-telomere assembly of Okinawan *O. dioica *genome sequences and give insight into the genomic diversity of *O. dioica* from different geographical locations. The data deposited in the EBI BioImage Archive provide representative images of the antibodies’ staining properties for use in epifluorescent and confocal based fluorescent microscopy.

 Introduction (paragraphs 1-3, complete). The larvacean, *Oikopleura dioica*, possesses a fascinating genome: it has reduced to a mere 70Mbp and exhibits unique characteristics such as non-canonical splicing and the scattering of Hox genes (Seo *et al.*, 2001; Edvardsen *et al.*, 2005; Marz *et al.*, 2008; Denoeud *et al.*, 2010). It is thought that a combination of large effective population size and high mutation rate per generation have led to fast evolution (Berná *et al.*, 2014). The recently published genome sequence of a “Japanese *O. dioica”* from mainland Japan highlighted large sequence variations between the Pacific and Atlantic populations (Wang *et al.*, 2020). In addition, we recently released a telomere-to-telomere genome sequence of an *O. dioica* individual collected from the Okinawan coastline in southern Japan (Bliznina *et al.*, 2020), which, to our surprise, revealed large differences in synteny to the mainland Japanese genome despite the geographical proximity. The genetic map of the north Atlantic *O. dioica* is reported to contain three chromosomes (two autosomes, X and Y sex chromosomes; Denoeud *et al.*, 2010); however, prior studies based on histochemical techniques reported three ( Körner, 1952) and eight chromosomes ( Colombera & Fernaux, 1973). Given the large sequence and synteny differences between the assembled *O. dioica*genomes, as well as the discrepancies among previous studies, we wished to assess the karyotype for the local Okinawan *O. dioica* population.

 Karyotyping is a long-established histochemical method to visualize eukaryotic chromosomes ( Hsu & Benirschke, 1967; Tjio & Levan, 1950). This rapid technique, involving the use of stains including methylene blue, eosin, and azure B, allows for observation of chromosomes with a simple light microscope, naturally lending itself to a first attempt for karyotyping analysis. However, we were unable to determine an accurate count for the Okinawan *O. dioica* by this method due to variability which ranged from 11-27 chromosomes per nucleus.

 As an alternative approach, we decided to immunostain the centromere as a means of quantifying the number of chromosomes. Metaphase-specific histone 3 (H3) markers have been used to determine the structure and the segregation of genetic material during oogenesis in situ ( Ganot *et al*., 2006; Schulmeister *et al.*, 2007). One such marker that has been successfully visualized in *O. dioica* is histone H3 phosphorylated at Ser-28 ( Kawajiri *et al.*, 2003; Kurihara *et al.*, 2006), whose localization depends on the phase of the cell cycle: during metaphase, sister chromatids were stained in a manner consistent with alignment along the metaphase plate, whereas in non-mitotic cells, spatially punctate signals were found evenly spread within the nuclear envelope ( Campsteijn *et al.*, 2012; Feng & Thompson, 2018; Feng *et al.*, 2019; Olsen *et al.*, 2018). A structure in which chromosomes are sequestered in a ∏-shaped conformation has also been observed during meiotic cell divisions between the final phases of oogenesis and mature oocytes ( Ganot *et al.*, 2008). In Table 1, we list the publications in which the H3S28P marker was applied to *O. dioica*: the studies were all performed using cultured strains originating from the north Atlantic Ocean. Here, we visualized anti-H3S28P stained embryos from two commercially available antibody sources and unfertilized oocytes to determine the chromosome count of the local Okinawan *O. dioica*.

 _____________________

**Reviewer 1 comment 1.2 –** **Misinterpretation of data**

**Reviewer comment**

 This piece of work can boost broad interests in using O. dioica as a new model in epigenetics and cell cycle studies. However, some results are a bit confusing to me and may be misinterpreted. 

**Author response** 

 We thank the referee for the detailed comments below. We agree that we misinterpreted some of the results and we have now revised the manuscript to correct this.

**Manuscript changes** 

 Specific instances of misinterpretations (response to comments 2.1 & 3.1) and changes in schematics have been addressed below (response to comments 2.2 & 3.3). Clarification of timing of oocyte collection has explained in more detail (response to comment 3.2).

 _____________________

**Reviewer 1 comment** **2.1** **–** **Cell cycle state of cells containing 12 spots**

**Reviewer comment**

 In Fig 1, centromere counts at prophase are 12, and at metaphase are 6, which are inconsistent. H3S28p signals locate at inner centromeric regions, flanked by CenpA signals that mark kinetochores at metaphase in embryonic mitosis in Norwegian *O. dioica*. The counts of H3S28p signals should be the same at prophase and metaphase, which are 6. 

**Author response** 

 We thank the reviewer for this comment. We agree that the cells containing ~12 spots  cannot be in prophase. In fact, we cannot explain the cell cycle state of these cells, so we now refer to them as “non-mitotic”.

**Manuscript changes** 

 Paragraph 12. “Cells were manually classified into two types depending on the staining pattern visible in the nucleus: (i) those with intense clusters of signals in the center, considered to be in metaphase and (ii) those containing evenly distributed, clearly separated spots within a faint background of signal defining a region encompassed by the nuclear envelope, interpreted as non-mitotic (Figure 1A and 1B, blue circles; Figure 1A and 1B, red squares). Counts from these two classes of nuclei fall into separate distributions (Figure 1C and 1D), with both epifluorescence and confocal acquisitions in agreement with each other. We interpreted the nuclei with an average of six large, clustered signals as centromeric regions in metaphase (Figure 1B), however, we cannot explain the cell cycle state of those containing the average of 12 spatially distinct punctate signals.”

 _____________________

**Reviewer 1 comment** **2.2** **–** **** **Schematic representation of chromosomes in embryos in Figure 1B**

**Reviewer comment**

 In addition, centromere is a piece of DNA sequence that holds a pair of sister chromatids in mitotic phase before they separate at anaphase. We can say that a chromosome has one centromere and a pair of sister chromatids at prophase.

 Thus, the schema representing prophase in Fig 1B should be a pair of sister chromatids is linked by one red dot at centromere.

**Author response** 

 We thank the referee for this comment. We have corrected our use of “centromere” and redrawn Figure 1B.

**Manuscript changes** 

 1. We have changed all instances of “a pair of centromeres” to “centromere”.

 2. Figure 1B. We have corrected the schematic representation of metaphase and non-mitotic nuclei in Figure 1B.   

 3. We have updated our manuscript to replace “ *centromere*” with “ *centromeric region*” when referring to the DNA sequence regardless of the state of assembly of the centromere, and removed mentions of “ *a pair of*” from the remaining occurences of “ *centromere*”. 

 _____________________

**Reviewer 1 comment** **3.1** **–** **Interpretation of H3S28P signal locations**

**Reviewer comment**

 H3S28P signals in female meiosis of Norwegian O. dioica are a bit different from those in mitosis. It localizes on entire chromosomes in prophase, moves towards centromeric regions during prometaphase, and is enriched at centromeric regions (or accurately speaking, midline of a bivalent) at metaphase I. Since chromosomes are more condensed in meiosis, and the midline of a bivalent should be crossover site between homologous chromosomes, we don’t know how far away it is between centromere and crossover in meiotic chromosomes of *O. dioica*, and how many crossovers a bivalent has. I would say centromeric region of H3S28p signals in female meiosis with caution. 

**Author response**

 We thank the referee for this comment. To make a clearer distinction between observation and interpretation, we now refer to the spots in the imaging data as “H3S28P signal” and only equate them to the centromeric region in specific instances. We have also included caveats to the interpretation of the oocyte data in the discussion section.

**Manuscript changes** 

 1. We now refer to the image spots as “H3S28P” signal, and only equate them to centromere in specific, appropriate contexts.

 2. Paragraph 12. “.... We interpreted the nuclei with an average of six large, clustered signals as centromeric regions in metaphase (Figure 1B), however, we cannot explain the cell cycle state of those containing the average of 12 spatially distinct punctate signals. ”

 3. Paragraph 13. “.... We interpreted each spot as representing a centromere from paired chromatids forming a synapsis in unfertilized eggs (Figure 2E).”

 4. Updated Paragraph 16-17.

 Paragraph 16. “An important consideration is what the H3S28P signal represents. It has been used to visualize centromeric regions in *O. dioica* (Table 1), but the signal is not confined to the centromere and its localization depends on the cellular state (Figure 1; Hake  et al., 2005; Feng and Thompson, 2018). However, we are confident that the signals seen in Figure 1 labelled as metaphase and Figure 2 represent centromeres and their associated chromosome. Further, DNA-staining images of mature oocyte have previously been interpreted as chromosomes condensed in a structure resembling the Greek character ∏ ( Ganot *et al.*, 2007). Since we did not perform DNA stains, our interpretation of the H3S28P signal in the oocyte does not preclude the previously reported ∏-structure. Additionally, the positions and numbers of crossovers between homologous pairs are unresolvable in this highly condensed state and the signal positions are not definitive of centromeric-regions.”

 Paragraph17. “Currently, the nucleotide sequence of the centromeric region is unknown for *O. dioica*, although chromatin immunoprecipitation with a H3S28P antibody followed by long-read sequencing might be able to provide this information. However, our whole embryo staining data (Figure 1) and the previous literature (Table 1) show that the H3S28P antibody produces non-centromeric signals which may confound such analysis. Thus, alternative targets such as other centromeric histone 3 variants (Moosmann *et al.*, 2011) might be preferable. Knowledge of centromeric sequences would also open the possibility of confirming these results with fluorescence *in situ*hybridization.”

 5. Figure 2 legend, last sentence. “… The positions of centromeric regions cannot be determined as chiasmata(s) are present along the homologous pairs of chromosomes in a highly condensed state.”

 _____________________

**Reviewer 1 comment** **3.2** **–** **Timing of oocyte collection**

**Reviewer comment**

 Actually, H3S28p shows several spots (more than 6) during prometaphase I, as can be seen Fig S4 in Feng and Thompson, 2018 *Cell Cycle *publication. The stages of meiosis depend on when the oocytes are collected. Just after spawning, the oocytes are before prometaphase I. Within 10 to 15 min after spawning, it is prometaphase I. Later, it should be at metaphase I. The timing of sampling is not indicated, which makes it even harder to interpret the data. 

**Author response** 

 We thank the referee for this comment. The process of rinsing the eggs took more than 15 min and so the oocytes were metaphase I. Changes were made in the methods section.

**Manuscript changes** 

 Paragraph 4. “Unfertilized eggs were treated similarly with three successive 10-minute washes.”

 Paragraph 6. “Washed eggs, 32 and 64 cell embryos (described above) were immediately fixed…”

 _____________________

**Reviewer 1 comment** **3.3** **–** **** **Schematic representation of chromosomes in embryos in Figure 2E**

**Reviewer comment**

 But again, it should one red dot between a pair of sister chromatids in Fig 2E. 

**Author response** 

 We thank the referee for this comment and we have corrected Figure 2E.

**Manuscript changes** 

 1. Figure 2E. Schematic corrected so there is one red spot between each pair of sister chromatid.
